# Predicting Thermal Resistance of Packaging Design by Machine Learning Models

**DOI:** 10.3390/mi16030350

**Published:** 2025-03-19

**Authors:** Jung-Pin Lai, Shane Lin, Vito Lin, Andrew Kang, Yu-Po Wang, Ping-Feng Pai

**Affiliations:** 1 Interdisciplinary Program of Education, National Chi Nan University, Nantou 54561, Taiwan; 2 Siliconware Precision Industries Co., Ltd., No. 123, Sec. 3, Dafeng Rd., Dafeng Vil., Tanzi Dist., Taichung City 42749, Taiwan; 3 Department of Information Management, National Chi Nan University, Nantou 54561, Taiwan; 4 PhD Program in Strategy and Development of Emerging Industries, National Chi Nan University, Nantou 54561, Taiwan

**Keywords:** machine learning, prediction, thermal resistance, packaging design

## Abstract

Thermal analysis is an indispensable aspect of semiconductor packaging. Excessive operating temperatures in integrated circuit (IC) packages can degrade component performance and even cause failure. Therefore, thermal resistance and thermal characteristics are critical to the performance and reliability of electronic components. Machine learning modeling offers an effective way to predict the thermal performance of IC packages. In this study, data from finite element analysis (FEA) are utilized by machine learning models to predict thermal resistance during package testing. For two package types, namely the Quad Flat No-lead (QFN) and the Thin Fine-pitch Ball Grid Array (TFBGA), data derived from finite element analysis, are employed to predict thermal resistance. The thermal resistance values include θ_JA_, θ_JB_, θ_JC_, Ψ_JT_, and Ψ_JB_. Five machine learning models, namely the light gradient boosting machine (LGBM), random forest (RF), XGBoost (XGB), support vector regression (SVR), and multilayer perceptron regression (MLP), are applied as forecasting models in this study. Numerical results indicate that the XGBoost model outperforms the other models in terms of forecasting accuracy for almost all cases. Furthermore, the forecasting accuracy achieved by the XGBoost model is highly satisfactory. In conclusion, the XGBoost model shows significant promise as a reliable tool for predicting thermal resistance in packaging design. The application of machine learning techniques for forecasting these parameters could enhance the efficiency and reliability of IC packaging designs.

## 1. Introduction

When electronic equipment operates, the temperature of its materials rises due to the consumption of electrical energy. Additionally, differences in the thermal expansion coefficients and shrinkage properties of the materials cause thermal stress in the jointed parts. This heat energy increases the temperature of components, including semiconductor products. Elevated temperatures can compromise the functionality, reliability, and safety of electronic equipment [1,2]. Therefore, it is crucial to ensure that electronic components are maintained at a stable temperature under any environmental conditions. Given the importance of heat dissipation performance in semiconductor packaging, the current trend in IC design favors increasingly smaller package sizes. As a result, thermal analysis has become an essential aspect of testing. Designers must accurately forecast the heat generated by semiconductor packaging during system applications, assess the heat dissipation performance of packaging materials and structures, and account for factors in the packaging design process.

Current thermal simulation methods for electronic products generally use finite element analysis to model airflow, temperature distribution, and heat transfer in IC packages, PCBs, electronic components, housings, and power electronic devices to provide accurate results [3]. However, these simulations may rely on assumptions that are inconsistent with realistic conditions. Additionally, the simulation method requires high-performance hardware, which increases the complexity of the simulation and, as a result, significantly raises the calculation time [4].

Due to the complexity of packaging design patterns, the combination of simulation experiments and machine learning can enable predictive capabilities using a minimal required training dataset. This approach is gradually being applied to solve thermal analysis challenges in packaging testing [5]. For example, Wang and Vafai [6] used the K-fold cross-validation algorithm and the support vector regression (SVR) algorithm to predict changes in the hotspot temperature of 3D wafers when modeling and cooling parameters are varied. Experiment results show that the prediction deviation is very small. Chen et al. [4] used different machine learning models to predict and analyze thermal resistance in packaged products, with results indicating that the artificial neural network model performed the best. Park et al. [7] employed three machine learning models to analyze the relationship between thermal flux and mechanical flux in semiconductor packages, verifying the results through finite element analysis of equivalent properties. Stoyanov et al. [8] used two different element model structures, namely multiple quadratic functions and neural networks, to predict thermal fatigue damage caused by temperature cycle loads in insulated gate bipolar transistor power electronic module bonding wires. The results confirmed that the proposed method and modeling technique provide finite element matching accuracy and effectively map highly non-linear spatial distributions of failure-related parameters. Kim and Moon [9] primarily used a deep neural network (DNN) model to estimate the effective thermal conductivity of flat heat pipes with various shapes and working conditions. Numerical results demonstrated that the DNN can accurately estimate effective thermal conductivity for different diffusion thermal resistances. Lai et al. [10] employed the Long Short-Term Memory (LSTM) method to efficiently predict reflow soldering temperature distributions, significantly reducing computation time. Wang et al. [11] used convolutional neural networks (CNNs) with Bayesian optimization to replace computational fluid dynamic simulations, accelerating the thermal optimization of multichip modules. Their technique improved computational efficiency, reduced chip junction temperatures, and enhanced manufacturing efficiency (Table 1).

This study aimed to use finite element simulation data and machine learning models to forecast the thermal resistance values and thermal characteristics of two IC package types, QFN and TFBGA. The rest of this study is organized as follows: Section 2 introduces the thermal resistance of the IC package. The proposed framework of forecasting thermal resistance is illustrated in Section 3. Numerical results are presented in Section 4. Conclusions are addressed in Section 5.

## 2. Thermal Resistance of IC Package

**Figure 1:**
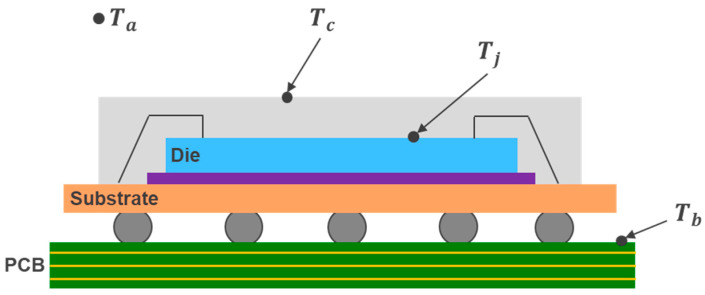
Critical temperature points of IC packaging.

The heat dissipation of semiconductor components occurs through two primary pathways: convection from the top of the package to the surrounding air and conduction from the package pins to the circuit board. The thermal resistance of an IC package is a key metric that measures the package’s ability to conduct heat generated by the die to the circuit board or the surrounding environment. By specifying the temperatures at two different points, thermal resistance quantifies the amount of heat flow between these two points. For thermal analysis of semiconductor packages, defining key temperature points of the package is essential. Figure 1 illustrates temperature points including Ambient Temperature (Ta), Junction Temperature (Tj), Case Temperature (Tc), and Board Temperature (Tb).

The thermal resistance of an IC package can be used to calculate the junction temperature of the IC based on power consumption and a given reference temperature. The thermal resistance $\theta_{jX}$ of heat flow from the junction point j to a specific temperature point x can be expressed as Equation (1).

(1) $$\theta_{jX}=\frac{T_{j}-T_{x}}{P}$$

where $T_{j}$ is the junction temperature, $T_{x}$ is the temperature at the specified location, and P is the total dissipated power.

The thermal characteristics of a typical IC package are shown in Table 2. Thermal resistance is an index indicating the temperature rise of the semiconductor product relative to the ambient temperature when the die generates one watt of heat. The measurement of thermal resistance is defined by the joint electron device engineering council (JEDEC). The thermal resistance θ_JA_ is defined for the IC package mounted on the test board and located in the surrounding environment. Since test environments and board designs vary significantly in many applications, θ_JA_ provides a thermal performance ranking of a package rather than simulating a specific end-use application. θ_JA_ is the thermal resistance of multiple heat dissipation paths that conduct heat transfers through conduction, convection, radiation, etc. θ_JB_ refers to the thermal resistance from the junction to the circuit board, including the thermal resistance from the junction of the IC to the reference point on the bottom of the package, and the thermal resistance through the circuit board at the bottom of the package. θ_JC_ represents the thermal resistance of the heat transfer path from the junction to a specific point on the top surface of the package case through specific conduction methods. The measurement of Ψ_JB_ is different from the direct single path in the measurement of thermal resistance θ_JB_. The measurement of Ψ_JB_ is based on multiple thermal paths, which represents the thermal characteristic parameters from the junction to the circuit board. Ψ_JT_ is a characteristic parameter that measures the temperature change between the junction temperature and the package top temperature.

There are various types of IC packages with different thermal conductivity characteristics. Using surface mount technology to connect PCBs, the QFN has become one of the most commonly used packaging types due to advantages in performance and cost. As the power demands of products continue to increase, the heat generated by the package also rises accordingly. The TFBGA is another common packaging type, characterized by a grid-like arrangement of pins on the surface. Package types of QFN and TFBGA generally provide high heat dissipation capability. Compared to the QFN, the TFBGA has the advantage of supporting more pins. Depending on the type and size of the packaging structure, internal temperatures can be significantly affected by varying thermal conduction interactions [12]. In packaging testing, numerical simulation and experimental methods are the most commonly used approaches to determine wafer packaging temperatures. However, both simulations and experiments for analyzing the thermal properties of packages are time- and resource-intensive [13].

## 3. Machine Learning in Predicting Thermal Resistance (MLPTR) Model

### 3.1. Machine Learning Models for Regression

Five machine learning models, namely support vector regression, multilayer perceptron, XGBoosting, light gradient boosting machine, and random forest, are employed to forecast thermal resistance in this study. LGBM is an ensemble learning model composed of multiple decision trees. Each tree is trained sequentially by residual errors of the previous trees to approximate the true values iteratively. The core mechanism of LGBM is its leaf-wise growth strategy, which selects the split that maximizes information gain at each step, leading to reduced error [14]. Additionally, LGBM employs histogram-based binning technology to convert continuous features into discrete intervals, thereby accelerating calculations. The objective of LGBM is to minimize the loss function. Random forest enhances prediction accuracy and model stability by aggregating the results of multiple decision trees. It demonstrates a strong capability to handle non-linear relationships, noise, and overfitting in data [15,16]. During the construction of each decision tree, the random forest employs random subsampling from the original training data to mitigate overfitting through random sampling. For each sample drawn from the training set, the random forest builds a decision tree and randomly selects a subset of features at each node, thereby reducing model bias. Once training is complete, the random forest combines all prediction results, leading to a finalized prediction value. XGBoost is based on gradient-boosted decision trees, which improves prediction results by gradually adding new trees [17]. Each time, the model is updated and is adjusted based on the prediction results of the previous model. A new tree is added to fit the difference between the predicted and actual values of the previous iteration. After the update, a new model is formed, and the most updated model is treated as the basis for the next round of learning. The goal of prediction is to make the predicted value close to the true value while maximizing the model’s generalization capability simultaneously. Therefore, this constitutes an optimization problem. While minimizing the difference between the predicted value and the target value, the loss function in the objective function is used to maximize the model’s fitness to the sample through continuous learning. XGBoost effectively manages overfitting through the regularization technique, supports distributed training, and facilitates feature importance analysis. As a result, XGBoost has become a popular alternative for regression tasks.

Support vector regression is developed for conducting regression within a certain allowable error range. SVR searches for a hyperplane with the smallest possible deviation from the training data and balances model generalization and accuracy. To achieve the aim mentioned, SVR minimizes the model’s complexity by controlling the deviation between the predicted and actual values within an acceptable range [18,19]. The main goal of SVR is to find the best regression line, ensuring the line is as close as possible to all data points while allowing some points to fall within the error range. A tube is used to allow prediction errors within a range of the SVR model. The weight vector controls the complexity of the model and provides a flatter decision function. When data points fall outside the acceptable deviation values, a penalty parameter is used to regulate the tolerance. Thus, the objective becomes minimizing the target function to find the optimal parameters [20,21]. The multilayer perceptron is a feedforward artificial neural network including an input layer, at least one hidden layer, and an output layer. Each neuron in a layer is connected to neurons in the subsequent layer through weighted connections, with computations incorporating non-linear activation functions [22,23]. An activation function is employed to decide whether a neuro is triggered or not. If a neuron is triggered, then a signal is conveyed forward to the neurons connected to the activated neuron. The MLP is trained using the backpropagation algorithm, which minimizes the loss function through gradient descent to reduce prediction error. Due to its capability of learning complex mappings, MLP is widely used in classification and regression tasks, making it a foundational component in deep learning.

### 3.2. Machine Learning Architecture for Thermal Resistance Prediction

**Figure 2:**
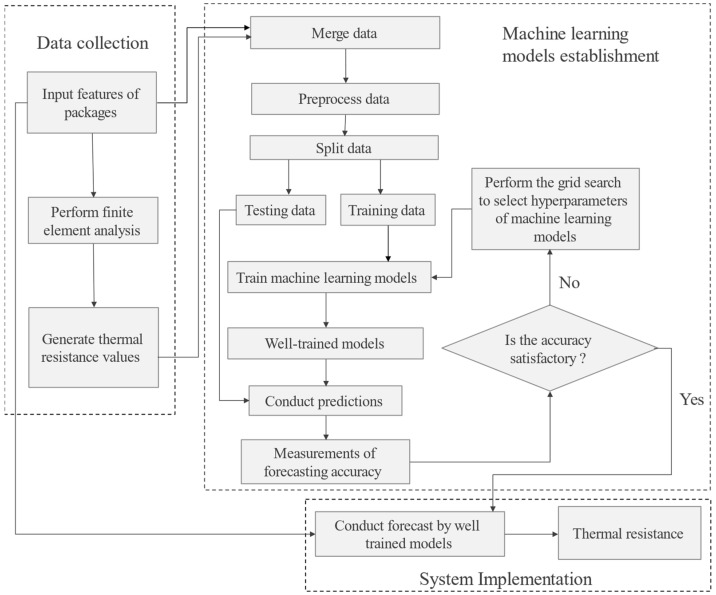
The proposed MLPTR framework.

To mitigate the time-consuming process using finite element analysis simulation, this study employs machine learning methods to predict thermal resistance. The framework of machine learning models in forecasting thermal resistance is shown in Figure 2. First, a dataset of simulated thermal resistance values was generated using finite element analysis. This dataset includes various input features related to the packaging structure, such as chip size and temperature.

Three stages, namely data collection, model establishment, and model implementation, are included in the developed machine learning in predicting thermal resistance (MLPTR) model. For the data collection stage, the features of the packages are fed into finite element analysis, and resistance values are generated. For the QFN package type with a total of 3234 data points, the dataset comprises twelve characteristic parameters including PKG Size, Die Size, Pad Size, Exposed Pad Size, Die Thickness, Mold Thickness, EMC K, Die K, Die Attach K, Leadframe K, Ambient Temperature, and input power. For the TFBGA package type, thirteen parameters, namely PKG Size, Die Size, Substrate Copper Ratio, Substrate Thickness, Die Thickness, Mold Thickness, EMC K, Die K, Thermal Via K, Die Attach K, BGA Ball, Ambient Temperature, and input power, are included with a total of 683 data points. Thermal resistance values, namely θ_JA_, θ_JB_, θ_JC_, Ψ_JT,_ and Ψ_JB_, are generated by finite element analysis and serve as target values in machine learning models. For the model establishment stage, the data are merged and invalid entries are removed. Then, the dataset is split into a training dataset and a testing dataset. The training dataset is employed for model learning, and the testing dataset is used for evaluating the performance of machine learning models. The numbers of training data points and testing data points for the QFN package type are 2587 and 647, respectively. For the TFBGA package type, 546 data points are for model training and 137 data points are for model testing. In this study, default values of hyperparameters are used for machine learning models, first for training models and then for conducting predictions. Then, measurements of forecasting accuracy are performed. When the forecasting accuracy is not satisfactory, the grid search algorithm is performed to select hyperparameters of the machine learning models. The final stage is an implementation phase. A well-trained machine learning model with the best forecasting accuracy is used to predict the thermal resistance practically.

## 4. Numerical Results

Five machine learning models, SVR, MLP, XGB, LGBM, and RF, are used to forecast thermal resistance in this study. Three techniques, including XGB, LGBM, and RF, are tree-based models. Two measurements, namely mean absolute percentage error (MAPE) and root mean square error (RMSE), are used to evaluate the forecasting accuracy of thermal resistance of the QFN package and the TFBGA package. MAPE and RMSE are calculated as shown in Equations (2) and (3).

(2) $$MAPE\;(\%)=\frac{100}{n}\sum_{i=1}^{n}\left|\frac{{\hat{Y}}_{i}-Y_{i}}{Y_{i}}\right|$$

(3) $$RMSE=\sqrt{\frac{\Sigma_{i=1}^{n}{\left({\hat{Y}}_{i}-Y_{i}\right)}^{2}}{n}}$$

where ${\hat{Y}}_{i}$ is the i-th predicted value, $Y_{i}$ is the i-th actual value, and i = 1~n.

**Figure 3:**
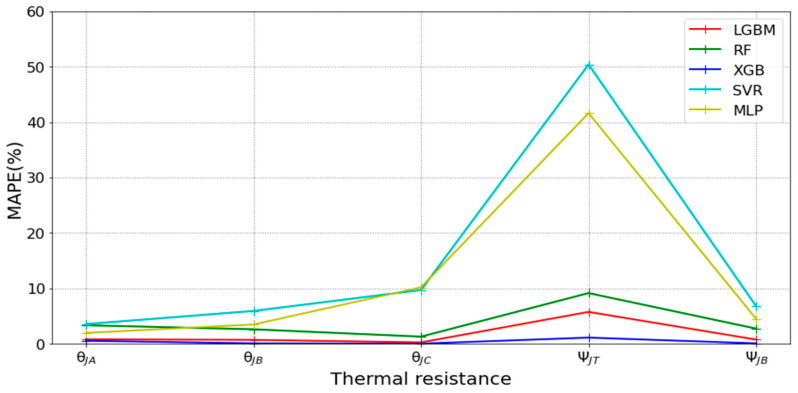
Visualization of MAPE (%) values for predicting the thermal resistance of the QFN package by machine learning models with hyperparameter selection.

**Figure 4:**
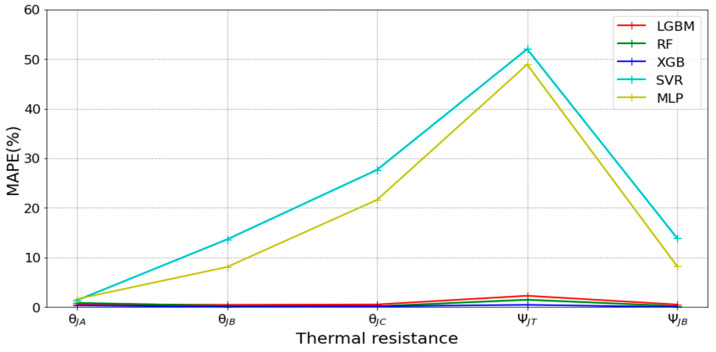
Visualization of MAPE (%) values for predicting the thermal resistance of the TFBGA package by machine learning models with hyperparameter selection.

**Figure 5:**
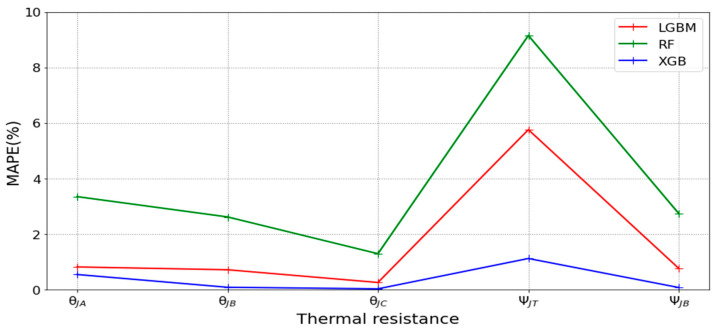
Visualization of MAPE (%) values for predicting the thermal resistance of the QFN package by tree-based models.

**Figure 6:**
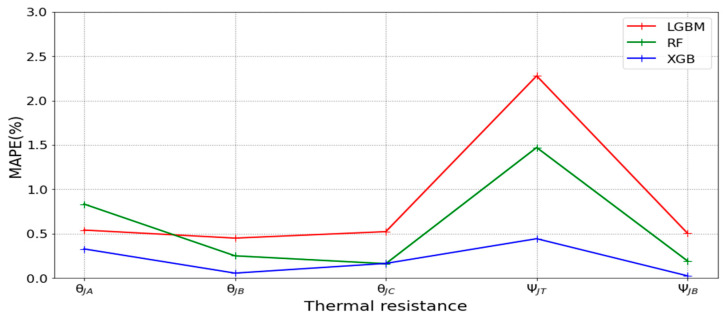
Visualization of MAPE (%) values for predicting the thermal resistance of the TFBGA package by tree-based models.

**Figure 7:**
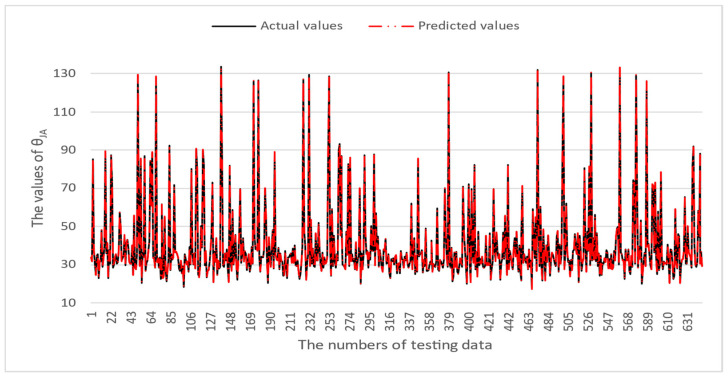
The actual values and predicted values generated by the XGB model of θ_JA_ for the QFN package type.

**Figure 8:**
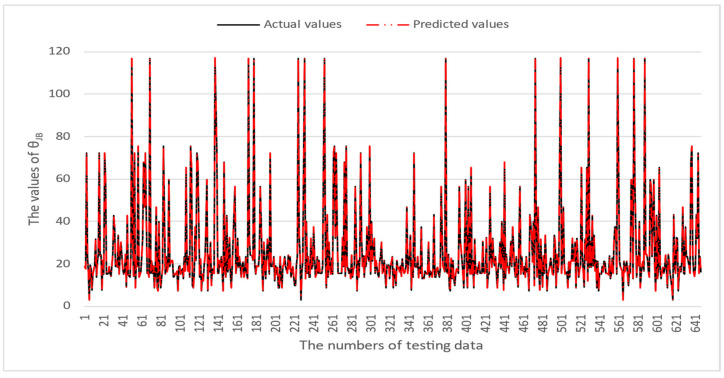
The actual values and predicted values generated by the XGB model of θ_JB_ for the QFN package type.

**Figure 9:**
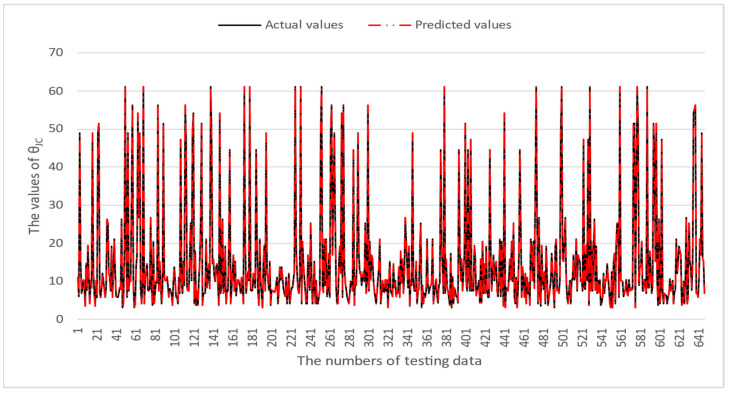
The actual values and predicted values generated by the XGB model of θ_JC_ for the QFN package type.

**Figure 10:**
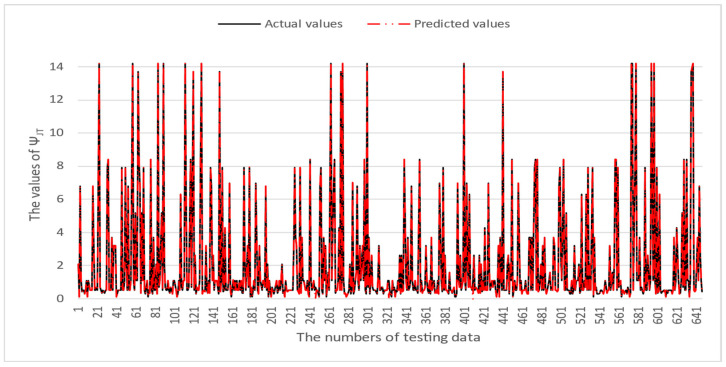
The actual values and predicted values generated by the XGB model of Ψ_JT_ for the QFN package type.

**Figure 11:**
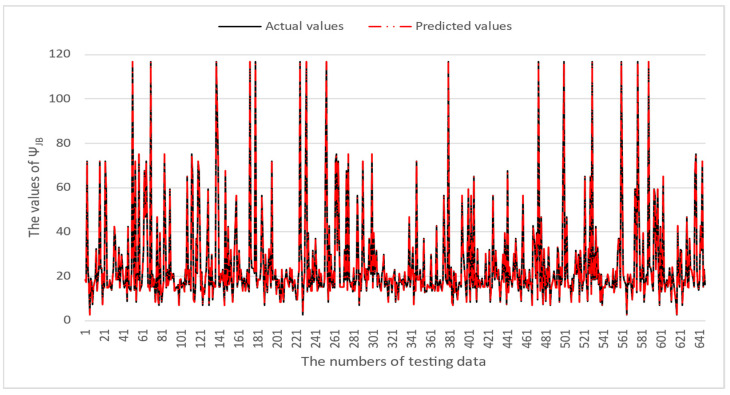
The actual values and predicted values generated by the XGB model of Ψ_JB_ for the QFN package type.

**Figure 12:**
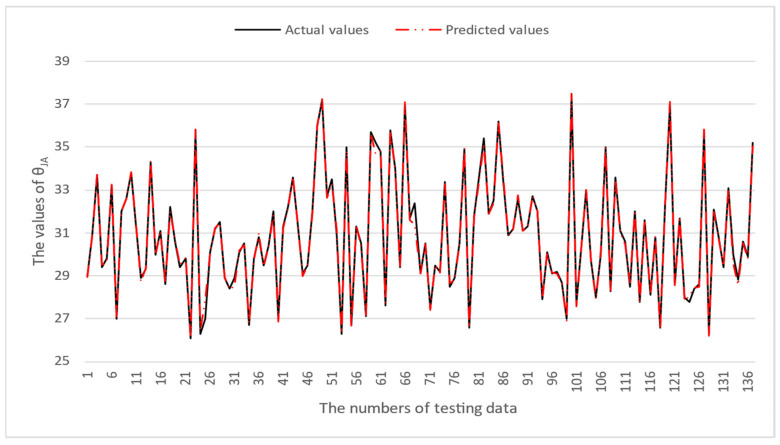
The actual values and predicted values generated by the XGB model of θ_JA_ for the TFBGA package type.

**Figure 13:**
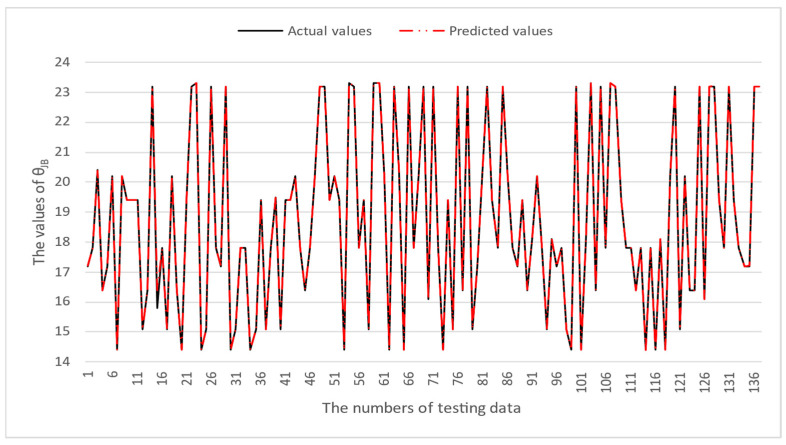
The actual values and predicted values generated by the XGB model of θ_JB_ for the TFBGA package type.

**Figure 14:**
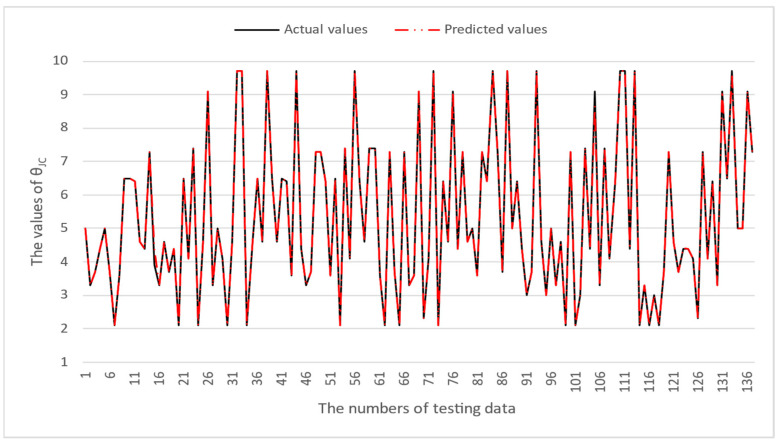
The actual values and predicted values generated by the XGB model of θ_JC_ for the TFBGA package type.

**Figure 15:**
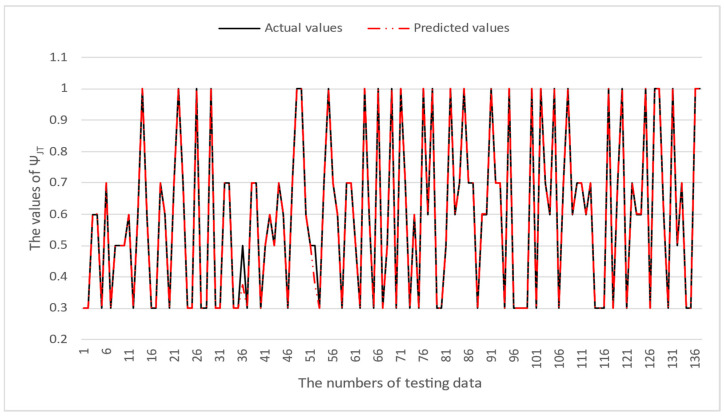
The actual values and predicted values generated by the XGB model of Ψ_JT_ for the TFBGA package type.

**Figure 16:**
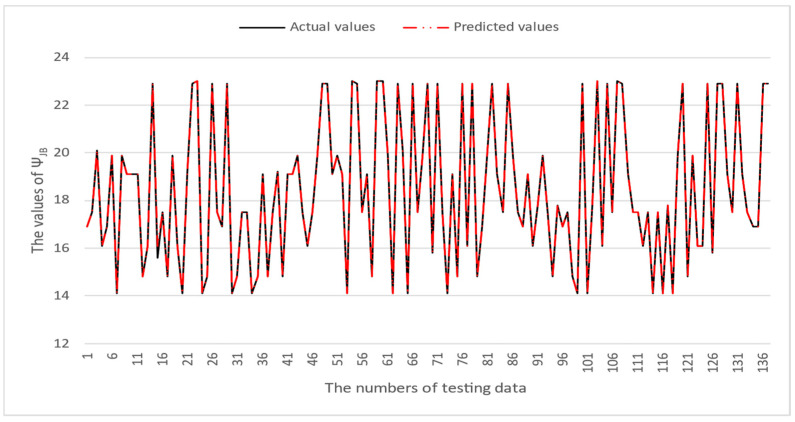
The actual values and predicted values generated by the XGB model of Ψ_JB_ for the TFBGA package type.

Table 3 and Table 4 illustrate the prediction performance of the QFN package and the TFBGA package by machine learning models. At this stage, default values are used for the hyperparameters. The MAPE and RMSE values indicate that the three tree-based models are superior to the SVR model and the MLP model in terms of forecasting accuracy. Notably, the SVR models and the MLP models are not able to capture trends of thermal resistance for the Ψ_JT_ package. To improve forecasting accuracy, the grid search technique was employed to select hyperparameters [24,25] for SVR models and MLP models. In this study, one hidden layer was employed for the MLP models. Table 5 lists the hyperparameters selected by the grid search algorithm for the two models. Table 6 indicates the forecasting performance of SVR and MLP using hyperparameters selected by the grid search algorithm. It can be observed that the selected hyperparameters improved the forecasting accuracy for both models. Figure 3 and Figure 4 illustrate the thermal resistance predictions with hyperparameter selection for the QFN package and the TFBGA package, respectively, in terms of MAPE (%) values. Furthermore, Figure 5 and Figure 6 reveal that the MAPE (%) values provided by the three tree-based models are less than 10% for both package types. The XGB model resulted in the best forecasting performance. The actual values and predicted values generated by XGB models of 10 packaging structures are illustrated in Figure 7, Figure 8, Figure 9, Figure 10 and Figure 11 for QFN package types and Figure 12, Figure 13, Figure 14, Figure 15 and Figure 16 for TFBGA package types.

## 5. Conclusions

This study uses machine learning models for effectively predicting thermal resistance in IC packaging processes. The forecasting results without hyperparameter selection indicate that tree-based models outperformed the SVR models and the MLP models in terms of MAPE (%) and RMSE values. For the three tree-based models, the XGBoost model generated the best forecasting results in both the QFN package and the TFBGA package. Then, the grid search algorithm was employed to determine the hyperparameters for the SVR models and the MLP models to improve forecasting performance. Numerical results indicated that the forecasting accuracy of the SVR models and the MLP models was increased by using the hyperparameter selection technique. However, the three tree-based machine learning models outperformed the SVR models and the MLP models with hyperparameter selection in terms of MAPE (%) values and RMSE values. In addition, the XBG models are superior to the other models in almost all cases. The superior predictive performance of XGB models is based on several factors. With regularization techniques to prevent overfitting and error correction capabilities during the learning process, XGB has been reported to perform well in forecasting tasks [26,27]. Additionally, Reddad et al. [28] highlighted that XGBoost is robust in predictive tasks due to its effective handling of parameter interactions, tree pruning, and hyper tuning. Studies [26,27,28] have successfully applied XGBoost to predict thermal conductivity, phase change material melting, and the reliability of solder ball joints. Incorporating data provided by the finite element analysis technique, this study concludes that the XGB model is a feasible, effective, and promising alternative for predicting thermal resistance in the IC package and testing industry. Additionally, the presented MLPTR framework could potentially be applied to address other regression problems in packaging design.

## Figures and Tables

**Table 1:** Thermal predictions of IC packaging by machine learning-based techniques.

Literature	Years	Applications	Methods
Chen et al. [4]	2024	Thermal resistance prediction of IC packages	ANN
Wang and Vafai [6]	2024	Predicting changes in hot-spot temperature on 3D wafers	SVR
Park et al. [7]	2024	Predicting thermal and mechanical flux in packages	SVR, GPR, ANN
Stoyanov et al. [8]	2024	Predicting thermal fatigue damage due to the temperature of power electronic modules	ANN
Kim and Moon [9]	2022	Estimating the effective thermal conductivity of a flat heat pipe	CNNs
Lai et al. [10]	2022	Predicting the reflow profile of a bulky ball grid array package	LSTM
Wang et al. [11]	2022	Optimizing the thermal layout of multi-die modules	CNN

Note: ANN—artificial neural network; GPR—Gaussian process regression.

**Table 2:** Symbols and descriptions of thermal characteristics.

Symbols	Descriptions
θ_JA_	Junction-to-Ambient Thermal Resistance
θ_JC_	Junction-to-Case Thermal Resistance
θ_JB_	Junction-to-Board Thermal Resistance
Ψ_JB_	Junction-to-Board Characterization Parameter
Ψ_JT_	Junction-to-Top Characterization Parameter

**Table 3:** MAPE (%) and RMSE values of machine learning models for predicting the QFN package’s thermal resistance.

	MAPE (%)				
	θ_JA_	θ_JB_	θ_JC_	Ψ_JT_	Ψ_JB_
LGBM	0.81910	0.71812	0.25985	5.76161	0.76677
RF	3.35084	2.61994	1.29613	9.15216	2.74191
XGB	0.54722	0.08679	0.03156	1.12312	0.07795
SVR	21.56125	38.94282	51.83620	74.77261	39.93139
MLP	9.44791	27.44147	54.38130	219.77778	28.19956
	RMSE				
	θ_JA_	θ_JB_	θ_JC_	Ψ_JT_	Ψ_JB_
LGBM	0.42701	0.18187	0.03496	0.08284	0.17618
RF	1.41133	0.59374	0.28861	0.31519	0.59424
XGB	0.29024	0.05605	0.00981	0.06757	0.02962
SVR	21.26226	21.61219	14.7613	3.34606	21.62768
MLP	5.87797	7.98745	7.08441	2.15471	7.84077

**Table 4:** MAPE (%) and RMSE values of machine learning models for predicting the TFBGA package’s thermal resistance.

	MAPE (%)				
	θ_JA_	θ_JB_	θ_JC_	Ψ_JT_	Ψ_JB_
LGBM	0.54050	0.45001	0.52274	2.27755	0.50401
RF	0.83332	0.25039	0.16172	1.47055	0.19218
XGB	0.32796	0.05541	0.16484	0.44334	0.02457
SVR	6.67386	14.03057	36.35358	46.45564	14.26818
MLP	3.83322	14.02184	24.36788	52.48518	12.89667
	RMSE				
	θ_JA_	θ_JB_	θ_JC_	Ψ_JT_	Ψ_JB_
LGBM	0.23402	0.10868	0.05360	0.01964	0.10976
RF	0.32222	0.10872	0.05480	0.02109	0.08745
XGB	0.16306	0.05172	0.05822	0.01496	0.04275
SVR	2.64245	3.03563	2.40439	0.25818	3.03495
MLP	1.39098	2.96866	1.40718	0.27544	2.68824

**Table 5:** Hyperparameters provided by the grid search for the SVR models and the MLP models.

		QFN					TFBGA				
Models	Hyperparameters	θ_JA_	θ_JB_	θ_JC_	Ψ_JT_	Ψ_JB_	θ_JA_	θ_JB_	θ_JC_	Ψ_JT_	Ψ_JB_
SVR	C	128	128	128	8	128	32	1	2	1	1
	ε	0.01	0.01	0.01	0.1	0.01	0.1	1.5	0.01	0.5	1.5
	gamma	0.1	0.1	0.1	0.1	0.1	0.1	0.1	0.1	0.1	0.1
MLP	Number of hidden nodes	150	150	50	100	50	100	150	100	50	100
	Activation functions	tanh	tanh	tanh	tanh	tanh	relu	relu	relu	relu	tanh
	Optimizers	adam	adam	adam	adam	adam	adam	adam	adam	adam	adam
	Learning rate	0.001	0.001	0.001	0.001	0.001	0.001	0.001	0.05	0.1	0.001

**Table 6:** MAPE (%) and RMSE values of SVR models and MLP models for predicting the thermal resistance of the QFN package and the TFBGA package with hyperparameter selection.

QFN	MAPE (%)				
	θ_JA_	θ_JB_	θ_JC_	Ψ_JT_	Ψ_JB_
SVR	3.55823	5.93015	9.70486	50.39072	6.77025
MLP	1.96832	3.48456	10.19002	41.61330	4.48553
	RMSE				
SVR	3.46385	3.35152	3.11810	1.98187	3.52001
MLP	0.89157	0.73737	1.25865	0.69823	0.88693
TFBGA	MAPE (%)				
	θ_JA_	θ_JB_	θ_JC_	Ψ_JT_	Ψ_JB_
SVR	1.34762	13.62841	27.65896	52.00729	13.86218
MLP	1.62015	8.07679	21.61422	48.88836	8.21445
	RMSE				
SVR	0.58975	2.91447	1.80322	0.26447	2.91400
MLP	0.64419	2.24097	1.36479	0.26156	2.24396

## Data Availability

The data presented in this study are available on reasonable request.
